# Aggregation‐Controlled Triplet Exciton Regulation Enables Single‐Emitter Color‐Evolving Afterglow

**DOI:** 10.1002/advs.77357

**Published:** 2026-08-26

**Authors:** Yu Zhang, Zonghang Liu, Gian Albert Alfani, Xilong Wei, Yue Zhang, Ziyu Cui, Yi Lu, Yixuan Chen, Dan Liu, Lin Lu, Zihao Deng, Shuyu Xue, Parvej Alam, Zheng Zhao, Ben Zhong Tang

**Affiliations:** ^1^ Guangdong Basic Research Center of Excellence for Aggregate Science School of Science and Engineering The Chinese University of Hong Kong (Shenzhen) Shenzhen Guangdong China; ^2^ Department of Chemistry and the Hong Kong Branch of Chinese National Engineering Research Center for Tissue Restoration and Reconstruction The Hong Kong University of Science and Technology Hong Kong China; ^3^ Institute of Materials Research Tsinghua Shenzhen International Graduate School Tsinghua University Shenzhen Guangdong China

**Keywords:** aggregation‐regulated emission, delayed fluorescence, information encryption, room‐temperature phosphorescence, single‐emitter afterglow, triplet exciton dynamics

## Abstract

Aggregation plays a crucial role in regulating excited‐state processes in organic luminescent materials, yet the simultaneous control of delayed fluorescence (DF) and room‐temperature phosphorescence (RTP) remains challenging. Herein, a series of dithienopyrrole‐based luminophores (DTP‐R) are developed as a single‐emitter system to investigate aggregation‐regulated excited‐state dynamics. By tuning the doping concentration in poly(methyl methacrylate) (PMMA), the emission evolves from monomer‐like to aggregate‐dominated states, accompanied by a continuous red shift of both prompt fluorescence (PF) and DF, as well as the coexistence of DF and RTP in the delayed spectra. Structural analysis reveals that aggregation is governed by cooperative weak intermolecular interactions, particularly S⋯π interaction, rather than conventional π‐π stacking. Theoretical calculations suggest that aggregation induces intermolecular charge separation, reduces the singlet‐triplet energy gap (ΔE_ST_), and provides more accessible triplet states, thereby favoring thermally activated RISC‐related DF. Temperature‐dependent delayed emission and ultralow‐concentration system DF further support a RISC‐mediated DF process. BPBr‐based host‐guest system regulates triplet‐exciton distribution through TTET, enhancing the phosphorescence contribution. As a single‐emitter system with concentration‐tunable color‐evolving afterglow, this system enables spatiotemporal information storage and dynamic anti‐counterfeiting, providing a strategy for designing organic afterglow materials with tunable excited‐state dynamics.

## Graphical abstract

A single‐emitter system based on DTP‐R luminophores enables time‐evolving afterglow through aggregation‐regulated excited‐state dynamics. The coexistence and suppression of delayed fluorescence and phosphorescence are controlled via concentration tuning and host‐guest engineering, revealing the RISC origin of delayed fluorescence and enabling spatiotemporal information storage.

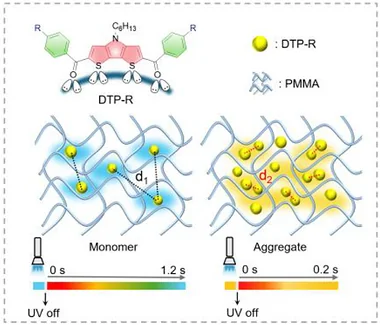

## 1 Introduction

The development of organic luminescent systems with time‐evolving afterglow has attracted significant attention owing to their potential applications in advanced information encryption, anti‐counterfeiting, and dynamic display [1, 2, 3]. In particular, afterglow color evolution introduces a temporal dimension to optical encoding, greatly enhancing information density and security [4, 5]. However, most reported systems rely on multiple emissive species, where energy transfer between different luminophores or the coexistence of distinct phosphorescence lifetimes (τ_p_) collectively leads to time‐dependent emission [6, 7, 8]. Although effective, such multi‐component strategies often suffer from compositional complexity, uncontrollable energy transfer, and limited tunability [9, 10, 11].

Achieving color‐evolving afterglow within a single emissive species remains highly challenging. This difficulty originates from the intrinsic competition between delayed fluorescence (DF) and room‐temperature phosphorescence (RTP), which arise from the same triplet exciton but follow fundamentally different relaxation pathways [12, 13, 14]. In most organic systems, either DF or RTP dominates, while their coexistence under ambient conditions is rare. More critically, even when both processes are present, controlling their temporal interplay to realize time‐dependent emission from a single emitter remains largely unexplored.

Aggregation provides a promising strategy to address this challenge [15, 16, 17]. By continuously modulating intermolecular interactions and electronic coupling without altering molecular structure, aggregation can regulate the singlet‐triplet energy gap (ΔE_ST_), charge‐transfer character, and non‐radiative decay pathways, thereby influencing the balance between DF and RTP [18, 19, 20]. Nevertheless, existing studies primarily focus on emission intensity or spectral shifts, while the role of aggregation in governing the dynamic competition between DF and RTP has not been fully established. Rather than ideal π‐π stacking, many systems are dominated by cooperative weak interactions, such as chalcogen bonding and hydrogen bonding, which can significantly influence excited‐state stabilization and decay pathways [21].

Beyond intrinsic aggregation effects, host‐guest engineering offers an external pathway to manipulate excited‐state dynamics [22, 23]. By introducing a phosphorescent host with suitable triplet energy levels, triplet excitons can be redistributed via triplet‐triplet energy transfer (TTET), thereby selectively suppressing or enhancing emissive channels [24]. Importantly, such systems provide a unique opportunity to probe exciton relaxation pathways through changes in τ_p_, offering direct mechanistic insight into whether delayed emission originates from reverse intersystem crossing (RISC) or alternative pathways such as triplet‐triplet annihilation (TTA) [25]. However, the combined role of aggregation and host‐guest interactions in regulating DF‐RTP competition and elucidating the origin of delayed emission remains largely unexplored [26].

Herein, we report a series of dithienopyrrole‐based luminophores (DTP‐R) that enable single‐emitter time‐evolving afterglow through aggregation‐regulated excited‐state dynamics. By dispersing DTP‐R into a rigid poly(methyl methacrylate) (PMMA) polymer matrix and tuning the doping concentration, a continuous transition from monomer‐like to aggregate‐dominated emission is achieved, accompanied by a fluorescence red shift exceeding 100 nm and the coexistence of DF and RTP. Structural analysis reveals that aggregation is governed by cooperative weak intermolecular interactions, particularly S⋯π contacts, rather than conventional π‐π stacking. Theoretical calculations demonstrate that aggregation induces intermolecular charge separation, reduces ΔE_ST_, and modulates RISC kinetics. Furthermore, host‐guest engineering with a phosphorescence‐only 4‐bromobenzophenone (BPBr) host enables TTET‐involved triplet‐exciton redistribution and enhances the phosphorescence contribution of DTP‐f. Combined with temperature‐dependent delayed emission and low‐concentration‐doped system DF observations, the host‐guest results support that the observed DF is more consistent with a thermally activated RISC process. This work establishes a general strategy for controlling excited‐state dynamics through the interplay of aggregation and host‐guest interactions, enabling single‐emitter spatiotemporally tunable afterglow. This single‐emitter platform reduces the compositional complexity of conventional multicomponent systems and shows potential for dynamic anti‐counterfeiting, time‐resolved information encryption, while providing new insights into triplet‐exciton dynamics in organic luminescent systems.

## 2 Results and Discussion

### 2.1 Molecular Design and Dual Delayed Emission in PMMA

**FIGURE 1:**
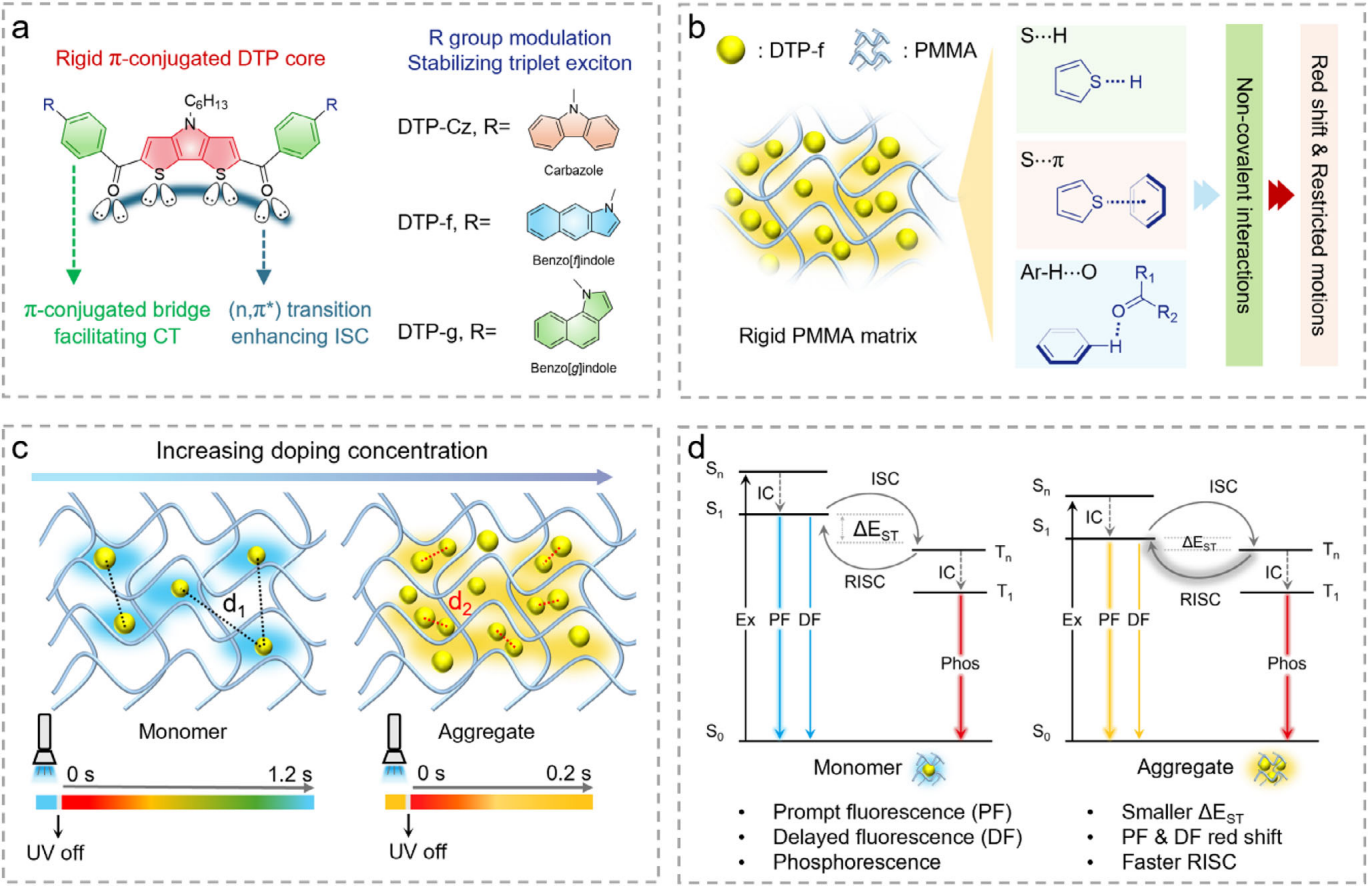
Molecular design and aggregation‐regulated excited‐state processes enabling color‐tunable prompt fluorescence (PF) and delayed dual emission of room‐temperature phosphorescence (RTP) and color‐tunable delayed fluorescence (DF). (a) Molecular design integrating multiple functional modules. The rigid π‐conjugated DTP core ensures efficient light absorption, while donor‐acceptor (D‐A) architecture promotes π‐bridge‐assisted charge transfer (CT). Meanwhile, (n,π*) transitions enhance intersystem crossing (ISC), facilitating efficient triplet exciton generation. (b) Schematic illustration of the rigidified environment in the PMMA matrix. The polymer host suppresses non‐radiative decay by restricting molecular motions, while intermolecular interactions (S⋯H, S⋯π, and Ar‐H⋯O) further stabilize the aggregate state and induce red‐shifted emission. (c) Concentration‐dependent aggregation behavior in PMMA. Increasing doping concentration reduces intermolecular distance (d_2_ < d_1_) and strengthens intermolecular interactions, leading to a gradual emission red shift and distinct afterglow evolution under UV (365 nm) irradiation and delayed fluorescence afterglow, corresponding to the emissive species from monomer to aggregate. (d) Schematic Jablonski diagram illustrates excited‐state processes at low and high doping concentrations. In both the monomer and aggregate states, reverse intersystem crossing (RISC) and triplet radiative decay collectively enable the coexistence of DF and RTP. In the aggregate state, the singlet energy level is lowered, resulting in a red‐shift of the fluorescence. (PF and DF).

A series of DTP‐R were designed by incorporating different donor fragments, where R denotes carbazole (Cz), benzo[*f*]indole (f), and benzo[*g*]indole (g), affording DTP‐Cz, DTP‐f, and DTP‐g, respectively (Figure 1a). This strategy enables systematic tuning of molecular electronic structures and intermolecular interactions, thereby providing a versatile platform to investigate aggregation‐regulated excited‐state processes (Figure 1b–d). As illustrated in Figure 1a, molecular design integrates several key considerations. The rigid π‐conjugated DTP core serves as the primary light‐absorbing scaffold, while the donor‐substituted benzoyl segments introduce intramolecular charge‐transfer (CT) character upon excitation. Meanwhile, the incorporation of heteroatom‐containing units provides lone‐pair (n) orbitals, enabling n→π* excited states. According to the El‐Sayed rule, intersystem crossing (ISC) is facilitated when the singlet‐triplet transition involves a change in orbital character [27]. As a result, both singlet and triplet excited states can be accessed, which is essential for enabling the coexistence of DF and RTP. In addition, the presence of multiple π‐conjugated segments and heteroatoms provides potential sites for intermolecular interactions, establishing the structural basis for aggregation‐dependent excited‐state dynamics. The synthetic routes toward the DTP‐R series are summarized in Scheme S1. The target compounds were fully characterized by ^1^H NMR, ^13^C NMR, and high‐resolution mass spectrometry (HRMS), and the corresponding spectra are provided in Figures S1–S17.

The DTP‐R series exhibits obvious aggregation‐dependent photophysical behavior, as evidenced by the distinct emission characteristics in different states. In dilute toluene solution (10^−5^ m, monomer), all compounds emit in the blue region at ≈ 460 nm, whereas in the solid powder (aggregate) state, a significantly red‐shifted emission peak at ≈ 580 nm is observed, corresponding to an aggregation‐induced spectral shift exceeding 100 nm (Figure S18). To bridge this large difference between monomer and aggregate states, DTP‐R molecules were dispersed into a rigid PMMA matrix, which serves as a controllable aggregation. The rigid environment effectively restricts molecular motions, suppresses non‐radiative decay, and minimizes quenching by oxygen and moisture [28]. Furthermore, the normalized UV–vis absorption spectra in different solvents (Figure S19) show only minor variations compared to the substantial emission shifts, indicating that the observed spectral evolution is primarily governed by excited‐state modulation rather than ground‐state electronic structure changes.

**FIGURE 2:**
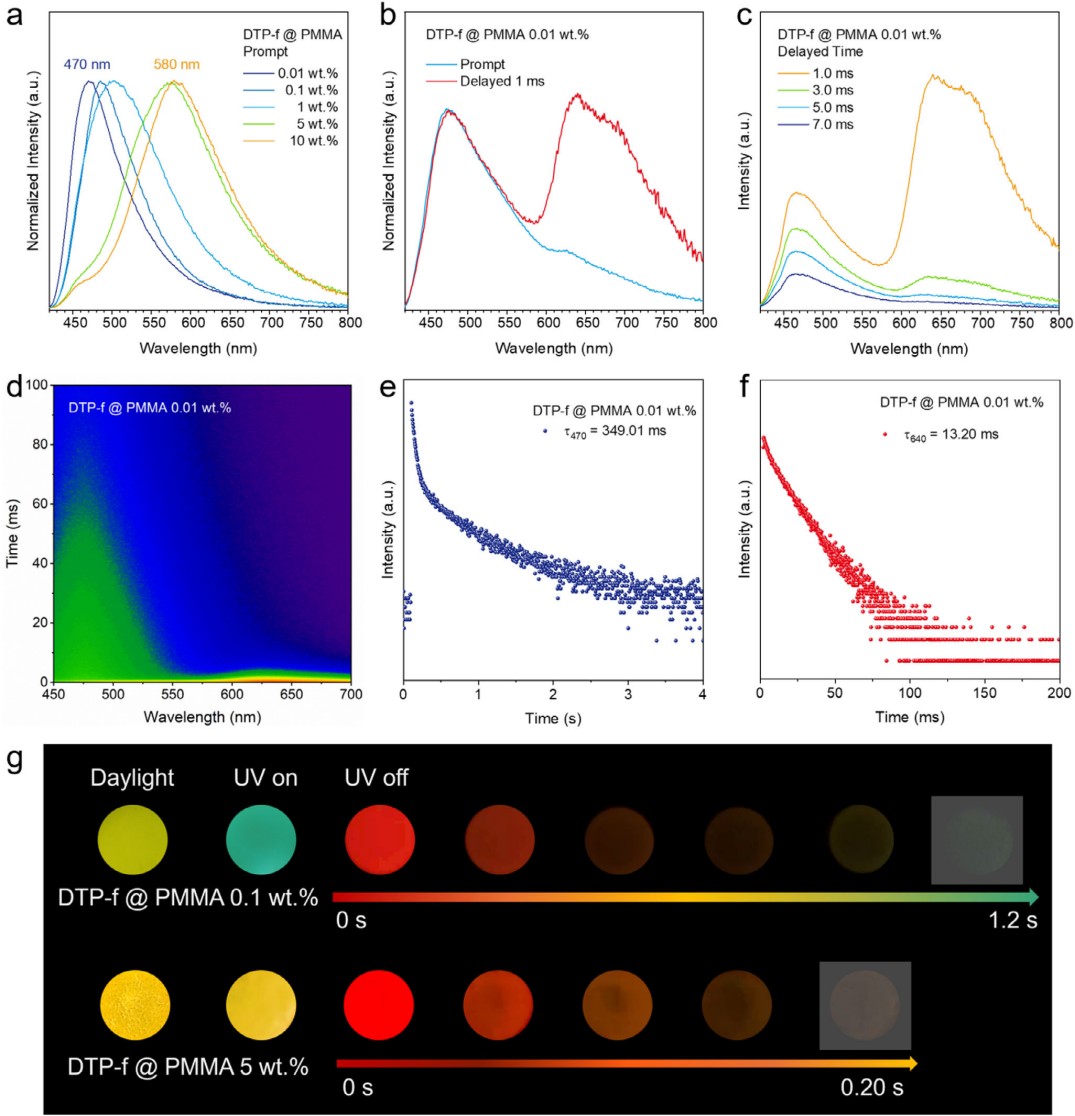
Photophysical properties and concentration‐dependent emission behavior of DTP‐f‐doped PMMA films. (a) Normalized steady‐state photoluminescence (PL) spectra of DTP‐f‐doped PMMA films at different doping concentrations (0.01–10 wt.%), showing a gradual red shift with increasing concentration due to enhanced intermolecular interactions stabilized lower singlet state in the aggregate state. (b) Steady‐state (prompt) and delayed PL spectra of DTP‐f‐doped PMMA film (0.01 wt.%, delay time = 1 ms). The delayed emission peak at ≈ 470 nm coincides with the PF, confirming that it originates from singlet‐state emission, assigned to DF, while the red emission band at ≈ 640 nm is assigned to RTP. c) Time‐resolved delayed PL spectra of DTP‐f‐doped PMMA film (0.01 wt.%) recorded at different delay times (1.0–7.0 ms). The emission at ≈ 470 nm (DF) shows slower decay, while the ≈ 640 nm emission (RTP) decays more rapidly, indicating distinct excited‐state relaxation pathways. (d) Time‐resolved emission spectra (TRES) mapping of DTP‐f‐doped PMMA film (0.01 wt.%), revealing the temporal evolution of dual emission, where the RTP decays rapidly while DF remains dominant at longer delay times. (e,f) Time‐resolved decay curve of DTP‐f‐doped PMMA film (0.01 wt.%) monitored at 470 and 640 nm, corresponding to DF and RTP, respectively. (g) Time‐dependent afterglow photographs of DTP‐f‐doped PMMA films at different doping concentrations (0.1 and 5 wt.%), showing concentration‐dependent afterglow color evolution.

As shown in Figure 2a, increasing the doping concentration from 0.01 to 10 wt.% results in a gradual red shift in the steady‐state photoluminescence (PL) spectra of DTP‐f‐doped PMMA films, reflecting enhanced intermolecular interactions in the aggregate state [29]. A similar concentration‐dependent PL evolution is observed for DTP‐Cz and DTP‐g (Figure S20), along with corresponding changes in CIE chromaticity coordinates, indicating that this behavior is a general feature of the DTP‐R series. Notably, among the three derivatives, DTP‐f exhibits superior miscibility in PMMA, as evidenced by its smooth and continuous spectral evolution toward the powder‐state emission, making it an ideal model system for mechanistic investigation. Control experiments confirm that pristine PMMA shows negligible emission under identical conditions (Figure S21), demonstrating that the observed optical responses originate exclusively from the doped luminophores.

The concentration‐dependent red shift indicates that increasing intermolecular proximity gradually stabilizes lower‐energy excited states through enhanced intermolecular interactions. This behavior is consistent with the model illustrated in Figure 1b,c, where the gradual reduction of intermolecular distance strengthens weak non‐covalent interactions, driving the emission from monomer‐like blue to aggregate‐state orange‐red. Among the three derivatives, DTP‐f was selected as the representative system for detailed spectroscopic analysis due to its superior miscibility and smooth spectral evolution. As shown in Figure 2b, the steady‐state and delayed PL spectra of DTP‐f‐doped PMMA film at 0.01 wt.% exhibit dual delayed emission (DF and RTP). In addition to a red emission band at ≈ 640 nm, assignable to RTP, the delayed spectrum also shows a blue emission band centered at ≈ 470 nm. Notably, this delayed spectra at ≈ 470 nm shows an identical emission peak and shape to the prompt fluorescence (PF), indicating that it originates from DF rather than higher T_n_ state phosphorescence. Similar overlap between prompt and delayed emission in the blue region is also observed for DTP‐Cz‐ and DTP‐g‐doped PMMA systems (Figures S22–S24), suggesting that the emergence of concentration‐dependent DF is a general feature of the DTP‐R series.

To understand the relaxation pathways of the two delayed emissive components, delayed PL spectra were recorded with delay times ranging from 1.0 to 7.0 ms (Figure 2c). The blue emission band at ≈ 470 nm persists over extended delay times, whereas the red emission at ≈ 640 nm decays much more rapidly, indicating distinct relaxation pathways. This difference is further visualized in the time‐resolved emission spectroscopy (TRES) mapping (Figure 2d), where the RTP component diminishes quickly while the DF gradually dominates at longer delay times. The corresponding lifetimes monitored at 470 and 640 nm (Figure 2e,f) yield lifetimes of 349.01 and 13.2 ms, respectively. Importantly, the afterglow characteristics also evolve systematically with concentration. As shown in Figure 2g, DTP‐f‐doped PMMA films at 0.1 and 5 wt.% exhibit distinct afterglow colors and decay times. Notably, the afterglow duration is significantly shortened at higher concentrations. This behavior suggests that aggregation influences excited‐state relaxation dynamics, where the accelerated decay may be associated with aggregation‐caused quenching (ACQ) or enhanced non‐radiative pathways [30]. Taken together, these results demonstrate that DTP‐R molecules in PMMA exhibit coexisting DF and RTP with distinct lifetimes, enabling time‐dependent afterglow color evolution within a single emissive system in different concentrations.

### 2.2 Structural Analysis of Intermolecular Interactions in Red‐Shifted Emission

**FIGURE 3:**
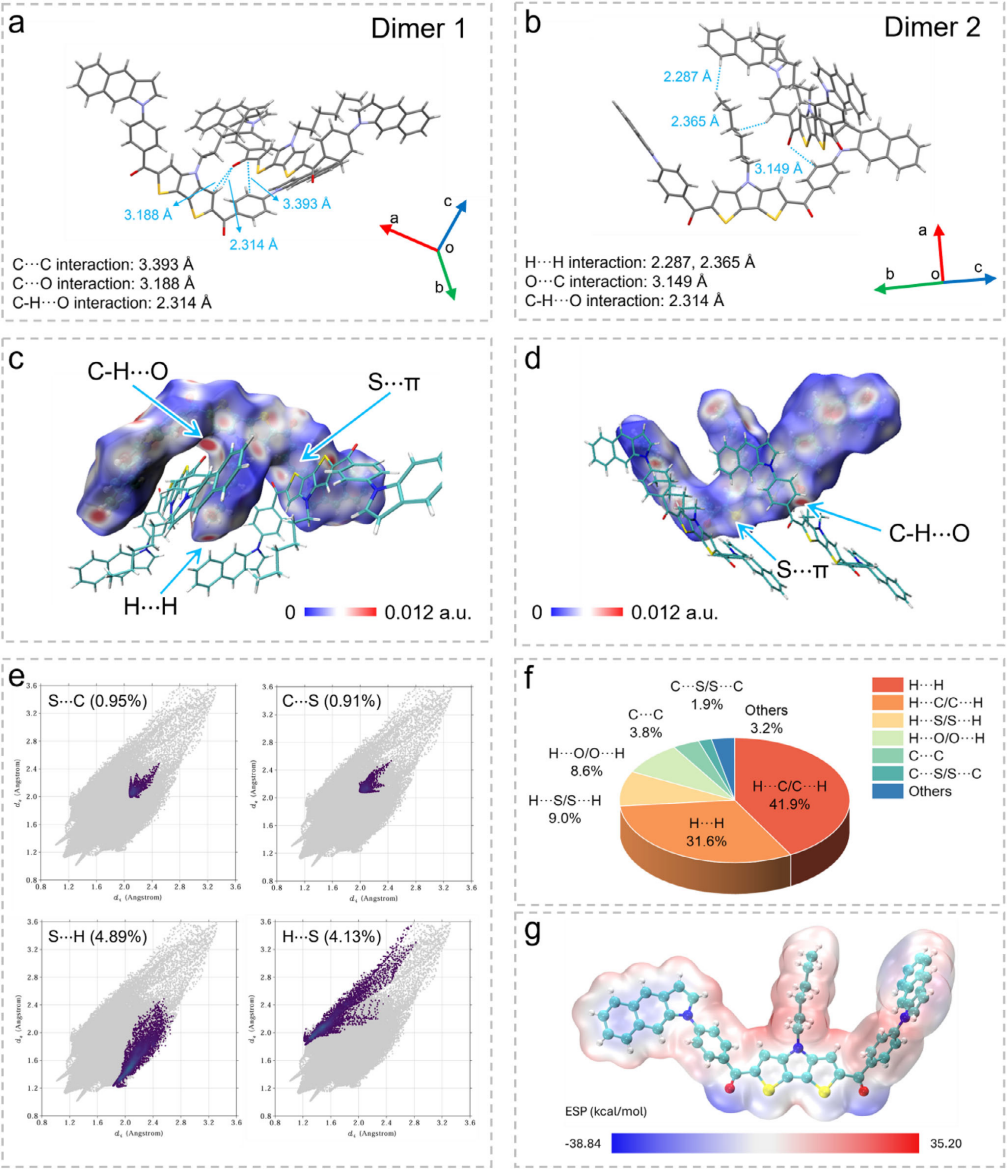
Intermolecular interaction analysis of DTP‐f from its crystal structure, combined with electrostatic surface potential (ESP) analysis, elucidates the electrostatic driving forces of these interactions. (a,b) Representative dimer configuration showing molecular packing mode and intermolecular distances of interactions. (c,d) Hirshfeld surface diagram revealing multiple non‐bonded interactions (S⋯π, H⋯H, and O⋯H contacts); red regions represent stronger interactions, and blue regions represent weaker interactions, respectively. (e) 2D Fingerprint plots of DTP‐f crystal quantifying the contributions of different intermolecular interactions of the sulfur atom (S⋯C, C⋯S, S⋯H, and H⋯S contacts). (f) Pie chart showing the percentage of individual atomic contacts, revealing that H⋯C/C⋯H and H⋯H interactions are dominant and play a key role in stabilizing the dimer formation. (g) ESP reveals the charge distribution and identifies potential interaction sites, thereby supporting the formation of multiple non‐bonded interactions.

To elucidate the origin of the aggregation‐induced red shift, single‐crystal analysis was undertaken, as the crystalline state represents a well‐defined aggregate system. All three DTP‐R single crystals exhibit orange‐red emission under UV irradiation (Figure S25), which is markedly red‐shifted relative to their blue emission in dilute toluene solution and low‐concentration PMMA films, indicating that the red‐shifted emission is intrinsically associated with aggregation. The crystal structure of DTP‐f is shown in Figure S26, and detailed packing information of DTP‐f is presented in Figure 3a,b, where a closely packed dimer with short intermolecular contacts is observed, suggesting that aggregate state photophysical behavior is governed by cooperative intermolecular interactions.

In many luminescent organic aggregates, red‐shifted emission is commonly attributed to efficient face‐to‐face π‐π stacking [31, 32, 33]. However, examination of the DTP‐f crystal packing suggests that such a conventional mechanism is not dominant in this system. The aromatic units do not adopt a typical parallel cofacial arrangement, but instead exhibit non‐parallel packing for the aromatic rings. This observation implies that alternative intermolecular interactions should be responsible for the observed red‐shifted emission. To figure out the nature of these interactions, Hirshfeld surface analysis was carried out using the Multiwfn program [34, 35]. As shown in Figure 3c,d, multiple effective contacts are present, including H⋯H, C‐H⋯O, and S⋯π interactions. The S⋯π contacts are of particular interest; compared with purely van der Waals contacts, such sulfur‐involved interactions are more likely to stabilize lower‐energy excited states. Therefore, the experimentally observed red shift is more plausibly associated with the cooperative effect of multiple weak intermolecular interactions, with S⋯π contacts playing a great important role. As shown in Figure 3e, the 2D fingerprint plots clearly resolve sulfur‐involved contacts, including S⋯C interactions associated with S⋯π coupling as well as S⋯H/H⋯S contacts, confirming the participation of sulfur in multiple intermolecular interactions. The corresponding percentage contributions (Figure 3f) indicate that H⋯C/C⋯H and H⋯H contacts dominate the overall interactions, primarily contributing to structural rigidification, whereas the smaller S⋯C contribution remains significant due to its direct relevance to emission red shift. Further insight into the origin of these interactions is provided by the electrostatic surface potential (ESP) surface (Figure 3g). The oxygen atom of the carbonyl group exhibits electron‐rich regions (−38.844 kcal/mol), which can interact favorably with electron‐deficient aromatic C═H sites (16.648∼25.932 kcal/mol), facilitating the formation of C═O⋯H contacts and stabilizing the dimer structure. Meanwhile, the sulfur atoms, owing to their high polarizability and positive electrostatic potential area (20.683 kcal/mol), can develop localized σ‐hole character [36, 37], enabling attractive interactions with electron‐rich π systems and thereby promoting S⋯π contacts.

Overall, the aggregate‐state stabilization arises from the cooperative effect of multiple weak non‐covalent interactions, where abundant contacts such as H⋯C/C⋯H and C═O⋯H primarily rigidify the packing framework, while sulfur‐mediated interactions play a more direct role in lowering the excited‐state energy. This synergistic interplay provides a structural basis for the aggregation‐induced red‐shifted emission observed in DTP‐f.

### 2.3 Host‐Guest Regulation of Triplet Exciton Relaxation Pathways

**FIGURE 4:**
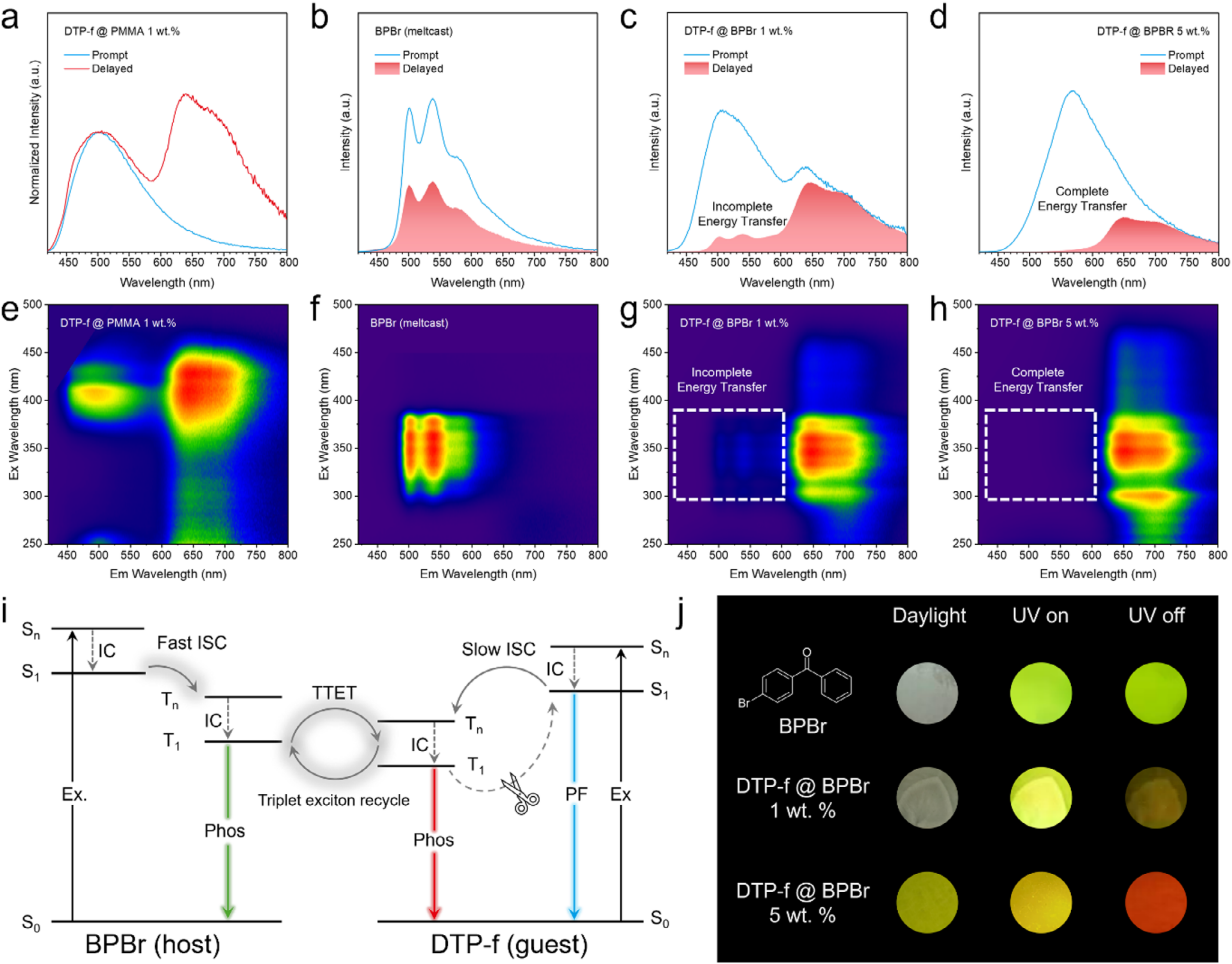
Photophysical properties and host‐guest energy transfer behavior of DTP‐f in 4‐bromobenzophenone (BPBr) matrix, compared with its intrinsic emission in PMMA. (a) Steady‐state and delayed PL spectra of DTP‐f‐doped PMMA film (1 wt.%), showing the intrinsic emission of DTP‐f in a rigid matrix, including PF, DF, and RTP. (b) Steady‐state and delayed PL spectra of melt‐cast BPBr, showing that the emission is dominated by phosphorescence with no detectable fluorescence component. (c,d) Steady‐state and delayed PL spectra of BPBr doped with DTP‐f (1 and 5 wt.%), showing enhanced phosphorescence from DTP‐f accompanied by a gradual suppression of BPBr triplet emission with increasing doping concentration. e‐h) Delayed excitation‐emission mapping of (e) DTP‐f‐doped PMMA film (1 wt.%), (f) melt‐cast BPBr, and BPBr doped with DTP‐f at (g) 1 wt.% and (h) 5 wt.%. The PMMA system (e) shows intrinsic delayed dual emission of DTP‐f with distinct excitation features, while BPBr (f) exhibits host‐dominated phosphorescence with characteristic excitation bands. In the host‐guest systems (g,h), the excitation features closely resemble those of BPBr, and enhanced phosphorescence is observed upon excitation within the BPBr excitation region, indicating that triplet excitons are predominantly generated from the BPBr host and subsequently transferred to DTP‐f via triplet‐triplet energy transfer (TTET). The gradual disappearance of the host emission region (dashed box) from (g) to (h) suggests more complete host‐to‐guest TTET with increasing dopant concentration. (i) Schematic Jablonski diagram illustrating the host‐guest excited‐state processes in DTP‐f‐doped BPBr. Triplet excitons generated in BPBr are transferred to DTP‐f via TTET due to energetically matched triplet levels, promoting exciton confinement within the triplet states while suppressing RISC, thereby diminishing delayed fluorescence. (j) Photographs under daylight, UV irradiation, and afterglow conditions, showing concentration‐dependent emission color and afterglow behavior of DTP‐f‐doped BPBr host‐guest system.

To reveal the origin of DF and further regulate triplet exciton relaxation pathways, a host‐guest strategy was employed by introducing BPBr as the host matrix. BPBr is a purely phosphorescent material featuring efficient ISC channels and long‐lived triplet excitons, enabling it to function not only as a rigid matrix but also as an effective triplet exciton reservoir capable of providing and redistributing triplet excitons [24, 38]. thereby altering their relaxation pathways and modulating the balance among PF, DF, and RTP. As a reference, DTP‐f‐doped PMMA film at 1 wt.% exhibits intrinsic dual delayed emission (DF and RTP) (Figure 4a), where PMMA serves only as a rigid matrix without external triplet excisions. In contrast, melt‐cast BPBr shows emission dominated exclusively by phosphorescence with no detectable fluorescence component (Figure 4b), confirming its role as an efficient phosphor and establishing a clear baseline for evaluating exciton transfer in the host‐guest system. Furthermore, the PL spectra of DTP‐Cz‐, DTP‐g‐, and DTP‐f‐doped BPBr at 0.1 wt.% (Figure S27) all show significantly enhanced RTP compared to their PMMA film, indicating the occurrence of host‐to‐guest TTET.

As shown in Figure 4c,d, increasing the DTP‐f concentration from 1 to 5 wt.% leads to a gradual disappearance of the characteristic emission features of BPBr, indicating that triplet excitons originally localized on the host are totally consumed by the guest. The overall photoluminescence quantum yield (PLQY) results further indicate that the total emission efficiency of the doped systems is mainly governed by the host matrix, while TTET from BPBr to DTP‐f increases the relative phosphorescence contribution of DTP‐f and thereby enhances the DTP‐f phosphorescence QY (Tables S1–S3). This process is further corroborated by the delayed excitation‐emission mappings (Figure 4e–h), where the PMMA film (Figure 4e) displays intrinsic dual emission with guest‐centered (DTP‐f) excitation features, while melt‐cast BPBr (Figure 4f) exhibits host‐dominated (BPBr) phosphorescence with distinct excitation bands; in contrast, the host‐guest systems (Figure 4g,h) show excitation band closely resembling those of BPBr, and enhanced phosphorescence of DTP‐f is observed upon excitation within the BPBr, indicating that triplet excitons are predominantly generated on the BPBr host and subsequently transferred to DTP‐f via TTET.

A key consequence of introducing BPBr is the complete suppression of DF, which provides critical insight into its origin. In the PMMA matrix, DTP‐f retains weak RISC channels, allowing a fraction of triplet excitons to repopulate the singlet state and generate a weak but detectable DF. However, in the BPBr host‐guest system, this pathway is significantly suppressed because the triplet energy levels of BPBr and DTP‐f are more favorably matched in both energy and spin character than the intrinsically spin‐forbidden return from the DTP‐f triplet state to its singlet state. As a result, triplet excitons preferentially remain within the triplet state and undergo host‐guest recycling rather than RISC, thereby leading to the disappearance of DF.

Additional kinetic evidence for this mechanism is provided by the phosphorescence decay behavior (Figure S28). In crystalline BPBr, the emission monitored at 500 and 550 nm corresponds to host‐centered phosphorescence with lifetimes of ∼19 ms. Upon doping DTP‐f at low concentration (1 wt.%), the host‐related lifetimes become slightly prolonged to 21 and 25 ms relative to pristine BPBr, indicating the emergence of additional slower relaxation pathways and supporting triplet exciton exchange or recycling between host and guest triplet states. In contrast, the longer‐wavelength emission bands at 650 and 700 nm, assigned to DTP‐f phosphorescence, exhibit significantly shorter lifetimes, demonstrating that the guest provides a faster radiative decay channel for triplet excitons. At higher doping concentration (5 wt.%), the characteristic BPBr emission at 500–550 nm disappears, and the decay becomes dominated by the shorter‐lived DTP‐f phosphorescence, indicating that triplet excitons are efficiently funneled to the guest and preferentially consumed through the faster guest‐centered phosphorescence pathway.

Based on these spectroscopic and kinetic results, a proposed Jablonski diagram is shown in Figure 4i. In this model, triplet excitons generated in BPBr transfer to DTP‐f through TTET, enabling dynamic triplet exciton recycling between host and guest at low dopant concentration, while gradually favoring guest‐centered phosphorescence as the DTP‐f content increases. Because this host‐guest triplet pathway is kinetically more favorable than RISC, excitons are effectively confined within the triplet state, thereby suppressing DF and enforcing triplet‐dominated decay in the BPBr matrix. These excited‐state changes are directly reflected in the photographs shown in Figure 4j, where concentration‐dependent emission color evolution and afterglow behavior are clearly observed. Therefore, the host‐guest strategy establishes deeper insight to reveal the relaxation pathways of DTP‐f.

### 2.4 Theoretical Analysis of Aggregation‐Regulated Excited‐State Dynamics

**FIGURE 5:**
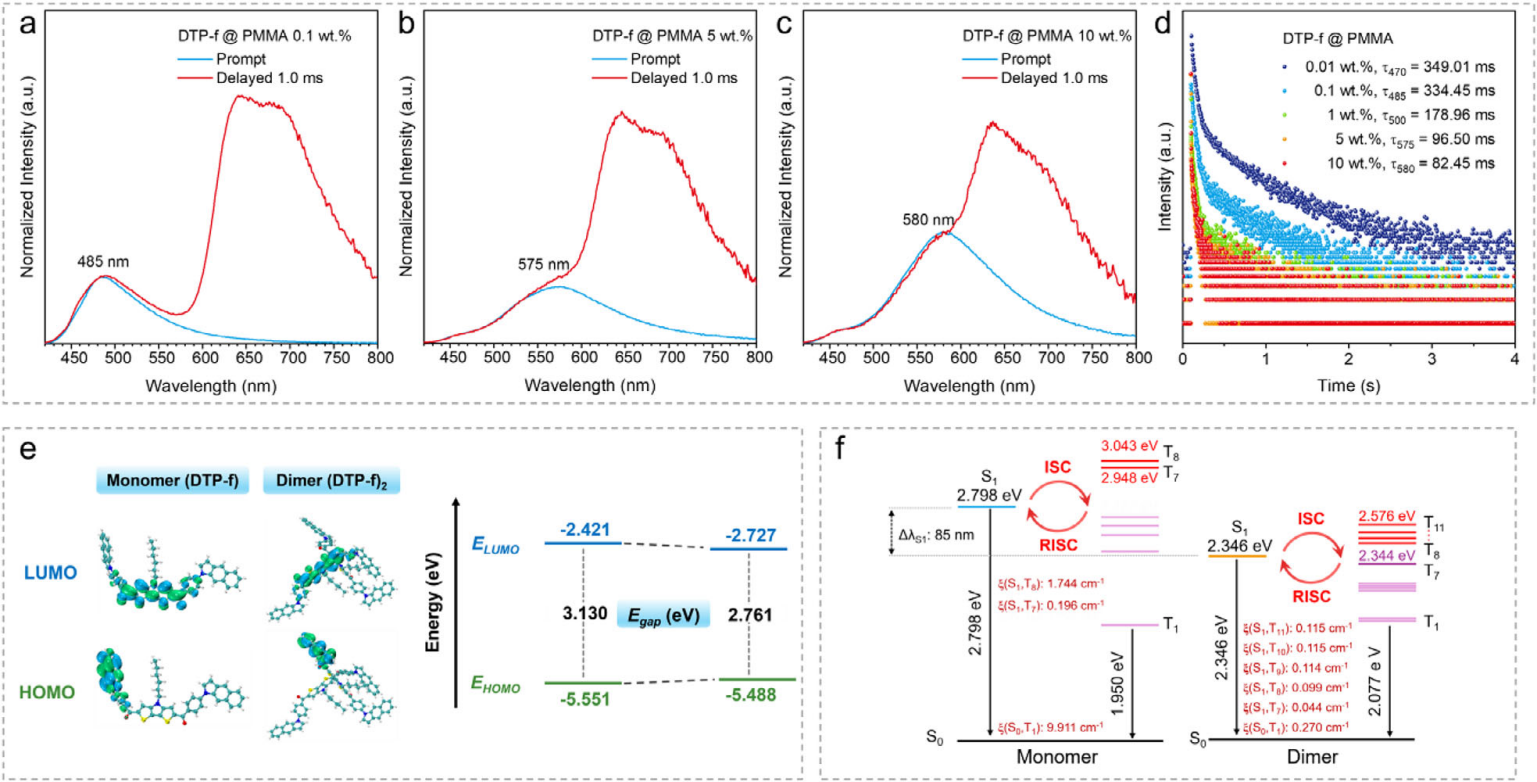
Photophysical mechanism and theoretical analysis of DTP‐f, revealing aggregation‐regulated excited‐state dynamics. (a–c) Steady‐state and delayed PL spectra of DTP‐f‐doped PMMA films at different doping concentrations (0.1‐10 wt.%), showing the coexistence of PF, DF, and RTP. A gradual red shift of fluorescence is observed with increasing concentration, due to enhanced intermolecular interactions stabilized lower singlet state in the aggregate state. (d) Time‐resolved decay curve monitored in the delayed fluorescence for DTP‐f‐doped PMMA films at different concentrations, showing shortened DF lifetimes (τ_DF_) with increasing doping concentration, suggesting accelerated decay pathways in the aggregate state. (e) Frontier molecular orbitals and energy levels of DTP‐f monomer (low concentration) and dimer (high concentration). The reduced energy gap (E_gap_) in the dimer originates from intermolecular electronic coupling in the aggregate state. The optimized structures are derived from crystal packing geometries. (f) Calculated excited‐state energy level diagrams and spin‐orbit coupling (SOC) constants of DTP‐f monomer and dimer. The dimer exhibits reduced singlet‐triplet energy gaps (ΔE_ST_) and increased density of triplet states; the experimentally observed shortening of τ_DF_ suggests that competing non‐radiative processes or intermolecular interactions dominate over RISC enhancement in the aggregate state. (The Marcus‐Levich theory equation shows the dependence of RISC rate (k_RISC_), SOC, and ΔE_ST_).

The origin of the concentration‐dependent emission red shift and the shortening of DF lifetime (τ_DF_) is clarified through a combined analysis of experimental observations and theoretical calculations. As shown in Figures 2a,b, 4a, and 5a–c, increasing the doping concentration of DTP‐f in PMMA leads to a continuous red shift of both PF and DF, indicating gradual stabilization of excited singlet state in the aggregate state. Meanwhile, the time‐resolved decay curves in Figure 5d and Figures S29–S31 reveal a gradual shortening of the τ_DF_ with increasing concentration, while τ_p_ remains essentially unchanged. This indicates that, during the aggregation process, the relaxation dynamics of triplet excitons distributed along the DF pathway undergo significant changes.

Insight into the origin of the red shift is obtained from theoretical calculations based on monomer and dimer models of DTP‐f, where the dimer geometry is extracted from crystal packing to ensure structural relevance to the aggregate state. Frontier molecular orbital (FMO) analyses shown in Figure 5e indicate that the monomer exhibits a typical intramolecular charge transfer, whereas the dimer shows intermolecular charge separation with frontier orbitals delocalized across adjacent molecules, demonstrating aggregation‐induced through‐space charge transfer. Correspondingly, the HOMO‐LUMO energy gap (E_gap_) decreases from 3.130 eV in the monomer to 2.761 eV in the dimer. In contrast, the calculated T_1_ to S_0_ transition characters show that both monomer and dimer retain dominant ^3^LE character (Figure S36), explaining why the RTP wavelength remains nearly unchanged upon aggregation. Further insight into DF dynamics is provided by excited‐state calculations using ORCA at the PBE0/def2‐SVP level with D3BJ correction (Figure 5f) for time‐dependent density functional theory (TD‐DFT) calculations. According to the Marcus‐Levich expression based on Fermi's Golden Rule [39, 40], the microscopic RISC rate is directly proportional to spin‐orbit coupling (SOC) and inversely proportional to ΔE_ST_ (Equation 1). To simplify the calculations, we will discuss only these two descriptors (SOC constant (ξ) and ΔE_ST_). Compared with the monomer, which exhibits relatively large SOC constants ξ(S_1_, T_8_) = 1.7440 cm^−1^ and ξ(S_1_, T_7_) = 0.196 cm^−1^ but relatively large ΔE_ST_, ΔE_S1T7_ = 0.150 eV, ΔE_S1T8_ = 0.245 eV, the dimer shows reduced SOC constants. Nevertheless, aggregation introduces a greater number of energetically accessible triplet states (T_7_‐T_11_) that can more effectively couple with S_1_ because of the smaller ΔE_S1Tn_. These additional triplet states possess smaller ΔE_ST_, which compensates for the reduced SOC and collectively facilitates more efficient RISC. For systems involving multiple thermally accessible triplet states, the overall RISC rate can be expressed as a Boltzmann‐weighted summation over individual channels (Equation 2) [41, 42]. In the present system, the increased density of accessible triplet states in the dimer provides additional thermally accessible RISC channels with smaller ΔE_ST_. Furthermore, the aggregation can lead to concentration quenching, which accelerates exciton deactivation. As a result, the τ_DF_ in the high‐concentration (dimer‐dominated) becomes shorter.

(1) $$\begin{array}{cc} & k_{T_{n}\to S_{1}}=\frac{2\pi }{\hbar }{\left|T_{n}\langle \left|{\hat{H}}_{SOC}\right|S_{1}\rangle \right|}^{2}\frac{1}{\sqrt{4\pi \lambda_{Tn\to S_{1}}k_{B}T}}\exp\\ & \times \left(-\frac{{\left(E_{S_{1}}-E_{T_{n}}+\lambda_{T_{n\to S_{1}}}\right)}^{2}}{4k_{B}T\lambda_{T_{n}\to S_{1}}}\right) \end{array}$$

(2) $$k_{RISC}=\sum_{n=1}^{\infty }\left(\frac{e^{-\frac{E_{T_{1}}}{k_{B}T}}}{e^{-\frac{E_{T_{1}}}{k_{B}T}}+e^{-\frac{E_{T_{n}}}{k_{B}T}}}\right)k_{T_{n}\to S_{1}}$$

where $k_{T_{n}\to S_{1}}$ is the transition rate from *T_n_* to *S*_1_; ℏ is the reduced Planck constant; $\left\langle T_{n}|{\hat{H}}_{\mathrm{SOC}}|S_{1}\right\rangle$ is the spin‐orbit coupling matrix element; $\lambda_{T_{n}\to S_{1}}$ is the reorganization energy; $E_{S_{1}}$ and $E_{T_{n}}$ are the energies of the singlet and triplet states, respectively; *k_B_* is the Boltzmann constant; *T* is the absolute temperature; and the Boltzmann factor in Equation (2) accounts for the thermal population of *T_n_*.

To further examine the delayed‐emission mechanism, temperature‐dependent delayed emission measurements were performed for DTP‐f‐doped PMMA at 1 wt.%. The short‐wavelength DF band becomes more pronounced at higher temperatures, while the long‐wavelength RTP band dominates at low temperatures and is progressively quenched upon heating (Figure S37a,b). The lifetime curve further shows different temperature responses of the DF and RTP components (Figure S37c,d). These results support the thermally activated nature of the DF component and indicate competition between DF and RTP pathways.

### 2.5 Single‐Emitter Time‐Evolving Afterglow Enabled Information Storage

**FIGURE 6:**
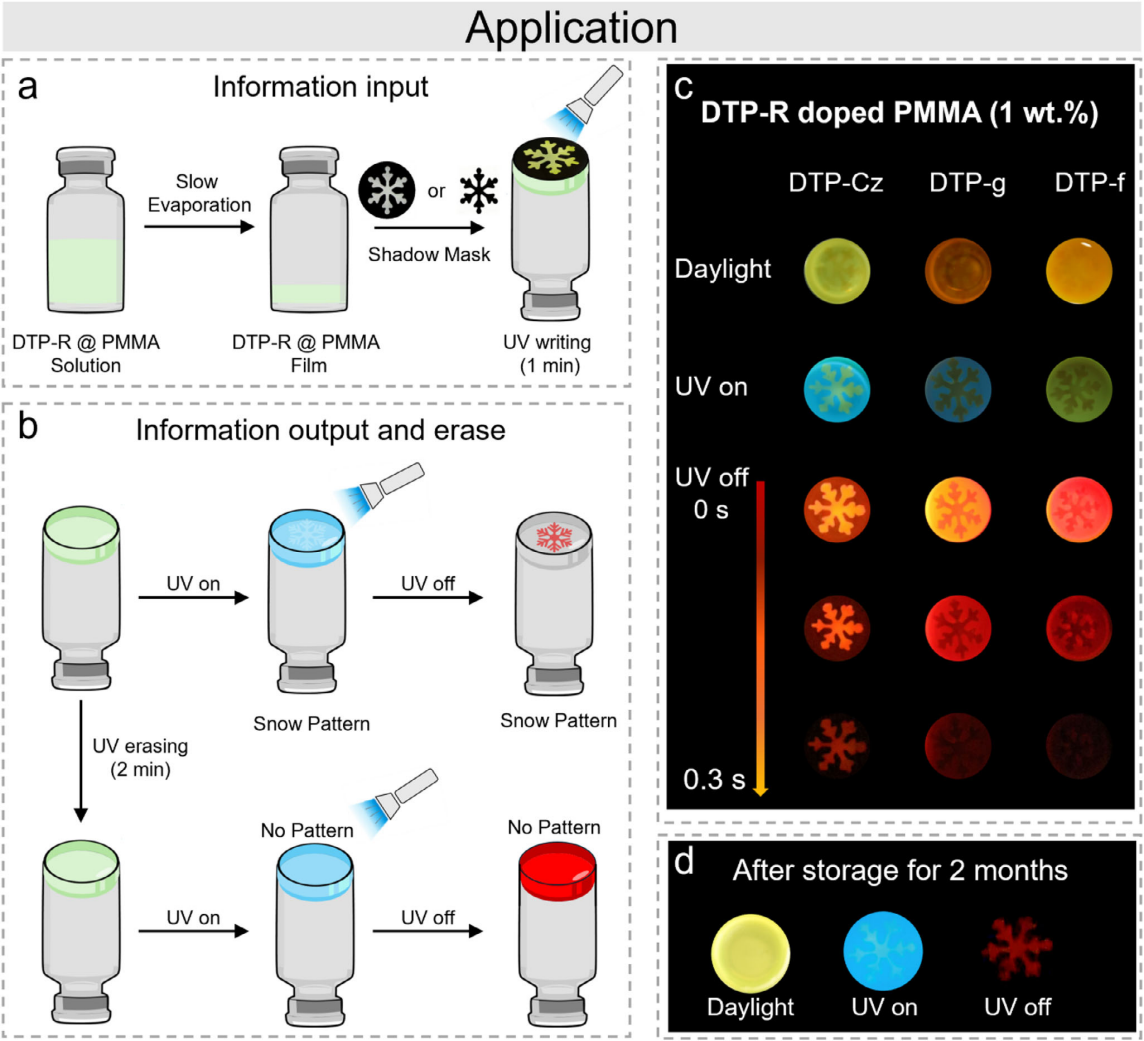
Application of DTP‐R‐doped PMMA films for information encryption. (a) Schematic illustration of DTP‐R PMMA film preparation and information writing process. DTP‐R‐doped PMMA films, followed by patterning using a shadow mask and UV irradiation to generate spatially defined emissive regions. (b) Schematic illustration of information readout and erasing processes. The stored information can be erased upon prolonged UV exposure without a shadow mask. (c) Photographs of DTP‐R‐doped PMMA films (1 wt.%) under daylight, UV irradiation, and afterglow conditions. (d) Stability tests of the patterned film after 2 months of storage showed that it has excellent information storage stability.

The coexistence of PF, DF, and RTP enables DTP‐R‐doped PMMA films to serve as versatile platforms for information encryption. As illustrated in Figure 6a, spatially resolved emissive patterns can be readily fabricated using a shadow mask under UV irradiation, allowing straightforward writing of information. Under continuous excitation, the patterns exhibit bright fluorescence for immediate readout, while upon removal of the excitation source, the emission evolves dynamically over time (Figure 6b). Although the DF contribution is relatively weak and difficult to resolve in practical readout, the dominant RTP channel provides a clear and tunable afterglow signal, enabling reliable temporal encoding. Furthermore, the three DTP‐R derivatives offer distinct emission colors and afterglow behaviors (Figure 6c), allowing a single spatial pattern to generate different visual outputs at different delay times, thereby increasing encoding complexity and information security. Importantly, the patterned films exhibit excellent stability, retaining their luminescence properties after storage for two months (Figure 6d). Overall, this system demonstrates that aggregation‐regulated excited‐state dynamics can be effectively translated into functional single‐emitter afterglow materials with potential applications in dynamic anti‐counterfeiting, time‐resolved information encryption, programmable security labels, optical data storage, and advanced display technologies.

## 3 Conclusion

In summary, three DTP‐R luminophores were synthesized and incorporated into a rigid PMMA matrix, where restricted molecular motion enables the coexistence of DF and RTP from a single emitter. By varying the doping concentration, the system evolves from monomer‐like to aggregate‐dominated states, accompanied by tunable PF and DF wavelengths and time‐dependent afterglow color. Structural analysis indicates that aggregation is governed by intermolecular interactions, particularly S⋯π contacts, rather than conventional π‐π stacking. Theoretical calculations show that aggregation induces intermolecular charge transfer, stabilizes the singlet excited state, reduces ΔE_ST,_ and increases the density of accessible triplet states, which facilitate RISC, while the shortened τ_DF_ arises from the combined effects of accelerated RISC and enhanced non‐radiative decay. Introducing the BPBr host further regulates triplet‐exciton distribution through TTET and enhances the phosphorescence contribution of DTP‐f. Together with the temperature‐dependent delayed emission and low‐concentration system DF behavior, these results indicate that the observed DF is more consistent with a thermally activated RISC process rather than TTA. These results demonstrate that aggregation and host‐guest interactions can be jointly used to modulate excited‐state dynamics, enabling single‐emitter time‐evolving afterglow and spatiotemporal information storage.

## Author Contributions

**Gian Albert Alfani**: investigation, visualization, methodology, data curation. **Xilong Wei**: investigation, data curation, methodology. **Yi Lu**: methodology, investigation. **Yixuan Chen**: methodology, investigation. **Ziyu Cui**: methodology, investigation. **Dan Liu**: methodology, investigation, validation. **Yu Zhang**: conceptualization, methodology, investigation, validation, visualization, project administration, writing – original draft, software, data curation. **Yue Zhang**: methodology, data curation, investigation. **Ben Zhong Tang**: conceptualization, methodology, supervision, funding acquisition, project administration, investigation, validation, visualization. **Parvej Alam**: supervision, conceptualization, methodology, software, funding acquisition, visualization, project administration, investigation, validation. **Lin Lu**: methodology, validation, investigation. **Zihao Deng**: methodology, validation, investigation. **Zonghang Liu**: software, visualization, writing – original draft. **Zheng Zhao**: conceptualization, methodology, supervision, funding acquisition, project administration, investigation, validation, visualization. **Shuyu Xue**: methodology, validation, investigation.

## Funding

This work was supported by the Major Program of the National Natural Science Foundation of China (Grant Nos. 22595400 and 22595401), the National Natural Science Foundation of China (Grant Nos. 52333007, 22305204, and 52273197), the Guangdong Basic Research Center of Excellence for Aggregate Science, the Science, Technology and Innovation Commission of Shenzhen Municipality (Grant No. KQTD20210811090142053), and theGuangdong Basic and Applied Basic Research Foundation (Grant No. 2023A1515110346).

## Conflicts of Interest

The authors declare no conflicts of interest.

## Supporting information




**Supporting File**: macp70113‐sup‐0001‐SuppMat.docx.

## Data Availability

The data that support the findings of this study are available in the supplementary material of this article.
