# Supplementary material for: Aggregation‐Controlled Triplet Exciton Regulation Enables Single‐Emitter Color‐Evolving Afterglow
Source: Adv Sci (Weinh). 2026 Aug 26:e77357. Online ahead of print. doi: 10.1002/advs.77357 (PMC13507911; doi:10.1002/advs.77357)
Supplement: Supplementary file 1 [file ADVS-9999-e77357-s001.docx]

Supporting Information

Aggregation-Controlled Triplet Exciton Regulation Enables Single-Emitter Color-Evolving Afterglow

*Yu Zhang, Zonghang Liu, Gian Albert Alfani, Xilong Wei, Yue Zhang, Ziyu Cui, Yi Lu, Yixuan Chen, Dan Liu, Lin Lu, Zihao Deng, Shuyu Xue, Parvej Alam*, Zheng Zhao*, Ben Zhong Tang**

Yu Zhang, Zonghang Liu, Gian Albert Alfani, Xilong Wei, Yue Zhang, Ziyu Cui, Yi Lu, Yixuan Chen, Dan Liu, Lin Lu, Shuyu Xue, Parvej Alam, Zheng Zhao, Ben Zhong Tang

Guangdong Basic Research Center of Excellence for Aggregate Science, School of Science and Engineering, The Chinese University of Hong Kong (Shenzhen), Longgang, Shenzhen, Guangdong 518172, China

E-mail: alamparvej@cuhk.edu.cn; zhaozheng@cuhk.edu.cn; tangbenz@cuhk.edu.cn

Lin Lu, Zihao Deng, Ben Zhong Tang

Department of Chemistry and the Hong Kong Branch of Chinese National Engineering Research Center for Tissue Restoration and Reconstruction, The Hong Kong University of Science and Technology, Clear Water Bay, Kowloon, Hong Kong 999077, China

Parvej Alam

Institute of Materials Research, Tsinghua Shenzhen International Graduate School, Tsinghua University, Shenzhen 518055, Guangdong, China

(Yu Zhang and Zonghang Liu contributed equally to this work.)

**Materials and Measurements**

All chemicals and reagents were purchased from commercial sources and used as received without further purification unless otherwise noted. Poly(methyl methacrylate) (PMMA) was purified prior to use to remove residual monomers, low-molecular-weight fractions, and additives. 2-Methyltetrahydrofuran (2-MeTHF) was purified by distillation to remove residual moisture and stabilizers. ^1^H NMR and ^13^C NMR spectra were recorded on a Bruker AVANCE NEO 500 MHz spectrometer using tetramethylsilane (TMS) as an internal standard. High-resolution mass spectra (HRMS) were obtained using a Waters Xevo G2-XS QTof mass spectrometer. Single-crystal X-ray diffraction (SC-XRD) data were collected on a Bruker single-crystal diffractometer, and the crystal structures were solved and refined using Olex2. Powder X-ray diffraction (PXRD) patterns were recorded on a Rigaku SmartLab diffractometer. UV-vis absorption spectra were measured using a PerkinElmer Lambda 365 UV-vis spectrophotometer. Steady-state photoluminescence (PL), delayed PL spectra, and time-resolved decay measurements were carried out on an Edinburgh Instruments FLS1000 spectrometer. Optical microscopy images of crystals were obtained using a Motic SMZ-161 microscope. Fluorescence and phosphorescence photographs were captured using a Canon camera.

**Computational Details**

All density functional theory (DFT) calculations were performed using Gaussian 16 software.^[1]^ The geometry optimization for hydrogen atoms was performed using the PBE0 functional with 6-311G** basis set, while the position for other element atoms were kept fixed.^[2]^ The Grimme’s D3 dispersion correction with Becke–Johnson damping was applied to account for long-range dispersion interactions.^[3]^ The frontier molecular orbitals (FMOs), electrostatic surface potentials (ESP), and Hirshfeld surface analysis were visualized using the VMD 1.9.3 program and Multiwfn 3.8.^[4,5]^ The spin–orbit coupling (SOC) matrix elements between the excited states were calculated by the TD-DFT at the PBE0-D3BJ/def2-SVP level using ORCA 5.0 program package. ^[6]^

**Synthesis and characterization**

**Scheme S1** Synthetic procedure of Lab Cz, DTP, DTP-Br, DTP-Cz, DTP-f, DTP-g.

**i: Synthesis of Lab-Cz (9*H*-carbazole)**

To a stirred solution of 2-nitro-1,1'-biphenyl (4.0 g, 20 mmol) in dry DMAc (60 mL) under a nitrogen atmosphere, triphenylphosphine (PPh_3_, 15.8 g, 60 mmol) was added. The reaction mixture was heated to reflux with vigorous stirring for 72 hours. Then, the mixture was cooled to room temperature, and the solvent was removed under reduced pressure. The crude residue was dissolved in CH_2_Cl_2_ and treated with activated carbon for decolorization. After stirring overnight, the mixture was filtered through a layer of diatomite to remove the activated carbon. The filtrate was concentrated and purified by column chromatography CH_2_Cl_2_/hexane (1:3, v/v), ethyl acetate/hexane (5:95, v/v), and CH_2_Cl_2_/hexane (1:2, v/v) as eluents successively. The obtained solid was further recrystallized, then redissolved in CH_2_Cl_2_ for single-crystal growth via CH_2_Cl_2_/petroleum ether vapor diffusion. Transparent colorless single crystals of Lab-Cz were obtained and used for next step synthesis (yield: 1.54 g, 45.8 %).

^1^H NMR (500 MHz, DMSO) δ 11.28 (s, 1H), 8.11 (d, J = 5.3 Hz, 2H), 7.50 (d, J = 7.9 Hz, 2H), 7.39 (t, J = 6.4 Hz, 2H), 7.16 (t, J = 6.8 Hz, 2H).

^13^C NMR (126 MHz, DMSO) δ 139.73, 125.52, 122.41, 120.17, 118.49, 110.95.

**ii: Synthesis of 4-hexyl-4H-dithieno[3,2-b:2',3'-d]pyrrole (DTP)**

In a dry 100 mL two-necked flask, 3,3'-dibromo-2,2'-bithiophene (2.50 g, 7.71 mmol), n-hexylamine (0.78 g, 7.71 mmol), Pd_2_(dba)_3_ (353.0 mg, 0.39 mmol), BINAP (961.4 mg, 1.54 mmol), and NaO*t*Bu (1.85 g, 1.28 mmol) were dissolved into 50 mL of toluene. The mixture was heated at 110 °C reflux for 24 h under a nitrogen atmosphere. After cooling to room temperature, the mixture was washed with brine water. The organic layer was dried over anhydrous Na_2_SO_4_. After the removal of the solvent, the crude material was purified by column chromatography (CH_2_Cl_2_/ Petroleum ether, 1/9 v/v) to afford a product as a white solid (yield: 1.45 g, 71.4%).

^1^H NMR (500 MHz, CD_2_Cl_2_) δ 7.15 (d, J = 5.3 Hz, 2H), 7.05 (d, J = 5.2 Hz, 2H), 4.21 (t, J = 7.1 Hz, 2H), 1.85 (q, J = 7.1 Hz, 2H), 1.34 - 1.25 (m, 6H), 0.87 (t, 3H).

^13^C NMR (126 MHz, CD_2_Cl_2_) δ 145.39, 123.06, 114.73, 111.52, 47.79, 31.81, 30.68, 27.01, 22.92, 14.16.

MS (TOF MS ES+): *m/z* calculated for C_14_H_17_NS_2_ [M]^+^: 263.0802; found: 263.0802.

**iii: Synthesis of DTP-Br**

In a dry 100 mL Schlenk tube, DTP (3.00 g, 11.39 mmol) was dissolved in CH_2_Cl_2_ (20 mL) under a nitrogen atmosphere. In a separate dry 250 mL two-necked flask, anhydrous AlCl_3_ (9.11 g, 68.33 mmol) and 4-bromobenzoyl chloride (10.00 g, 45.56 mmol) were dissolved in CH_2_Cl_2_ (40 mL) and cooled to 0 °C under nitrogen. After stirring for 15 min, the DTP solution was added dropwise over 15 min. The reaction mixture was then heated to 40 °C and refluxed for 72 h. Upon completion, the reaction mixture was cooled to room temperature and quenched with ice water. The organic layer was separated, washed with water, and dried over anhydrous Na_2_SO_4_. After removal of the solvent under reduced pressure, the crude product was purified by column chromatography on silica gel using CH_2_Cl_2_/petroleum ether (1:1, v/v) as the eluent to afford a yellow solid. Yield: 3.54 g (49.4%).

^1^H NMR (500 MHz, CD_2_Cl_2_) δ 7.76 (d, *J* = 8.1 Hz, 4H), 7.70 (d, *J* = 8.1 Hz, 4H), 7.56 (s, 2H), 4.22 (t, *J* = 7.0 Hz, 2H), 1.83 (p, *J* = 7.2 Hz, 2H), 1.32 - 1.21 (m, 6H), 0.84 (t, *J* = 6.8 Hz, 3H).

^13^C NMR (126 MHz, CD_2_Cl_2_) δ 187.83, 147.63, 143.56, 137.47, 132.19, 130.94, 127.21, 121.93, 118.58, 47.87, 31.68, 30.60, 27.00, 22.88, 14.12.

MS (TOF MS AP+): *m/z* calculated for C_28_H_23_Br_2_NO_2_S_2_H [M+H]^+^: 629.9596; found: 629.9594.

**iv: Synthesis of DTP-Cz**

In a dry 100 mL two-necked flask, DTP-Br (1.00 g, 1.59 mmol), 9*H*-carbazole (0.79 g, 4.77 mmol), Pd_2_(dba)_3_ (72.7 mg, 79.44 µmol), (*t*-Bu)_3_PHBF_4_ (91.2 mg, 317.75 µmol), and NaO*t*Bu (458.1 mg, 4.77 mmol) were dissolved in 20 mL of toluene under a nitrogen atmosphere. The reaction mixture was refluxed at 110 °C for 48 hours. After cooling to room temperature, the mixture was washed with brine. The organic layer was then extracted three times with ethyl acetate, and the combined organic extracts were dried over anhydrous Na_2_SO_4_. After solvent removal, the crude product was purified by column chromatography (CH_2_Cl_2_ / Petroleum ether, 1/1 v/v) to yield the product as an orange solid (0.57 g, 44.9%).

^1^H NMR (500 MHz, CD_2_Cl_2_) δ 8.18 (d, *J* = 5.1 Hz, 8H), 7.82 (d, *J* = 8.1 Hz, 4H), 7.76 (s, 2H), 7.59 (d, *J* = 8.2 Hz, 4H), 7.48 (t, *J* = 7.7 Hz, 4H), 7.35 (t, *J* = 7.4 Hz, 4H), 4.33 (t, *J* = 7.0 Hz, 2H), 1.93 (p, 2H), 1.36 - 1.26 (m, 6H), 0.84 (t, *J* = 6.9 Hz, 3H).

^13^C NMR (126 MHz, CD_2_Cl_2_) δ 187.88, 147.76, 143.87, 141.72, 140.74, 137.14, 131.24, 126.98, 126.60, 124.13, 121.97, 120.94, 120.78, 118.61, 110.18, 47.95, 31.73, 30.68, 27.03, 22.90, 14.16.

MS (TOF MS AP+): *m/z* calculated for C_52_H_39_N_3_O_2_S_2_H [M+H]^+^: 802.2562; found: 802.2558.

**v: Synthesis of DTP-f**

In a dry 100 mL two-necked flask, DTP-Br (1.00 g, 1.59 mmol), 1*H*-benzo[*f*]indole (0.79 g, 4.77 mmol), Pd_2_(dba)_3_ (72.7 mg, 79.44 µmol), (*t*-Bu)_3_PHBF_4_ (91.2 mg, 317.75 µmol), and NaO*t*Bu (458.1 mg, 4.77 mmol) were dissolved in 20 mL of toluene under a nitrogen atmosphere. The reaction mixture was refluxed at 110 °C for 48 hours. After cooling to room temperature, the mixture was washed with brine. The organic layer was then extracted three times with ethyl acetate, and the combined organic extracts were dried over anhydrous Na_2_SO_4_. After solvent removal, the crude product was purified by column chromatography (CH_2_Cl_2_ / Petroleum ether, 1/1 v/v) to yield the desired product as a reddish solid (0.30 g, 23.6%).

^1^H NMR (500 MHz, CD_2_Cl_2_) δ 8.21 (d, *J* = 9.0 Hz, 4H), 8.15 (d, *J* = 8.5 Hz, 4H), 7.98 (d, *J* = 7.0 Hz, 2H), 7.93 (d, *J* = 7.2 Hz, 2H), 7.85 (d, *J* = 8.5 Hz, 4H), 7.74 (s, 2H), 7.70 (d, *J* = 3.5 Hz, 2H), 7.44 - 7.37 (m, 4H), 6.92 (d, *J* = 4.4 Hz, 2H), 4.33 (t, *J* = 7.1 Hz, 2H), 1.92 (p, *J* = 7.0 Hz, 2H), 1.36 - 1.25 (m, 6H), 0.85 (t, *J* = 7.0 Hz, 3H).

^13^C NMR (126 MHz, CD_2_Cl_2_) δ 187.59, 147.68, 143.87, 143.73, 136.21, 135.73, 131.67, 131.47, 131.31, 131.06, 129.67, 128.42, 128.03, 124.73, 123.81, 123.43, 121.82, 119.22, 118.43, 106.67, 104.99, 47.93, 31.76, 30.69, 27.07, 22.93, 14.19.

MS (TOF MS AP+): *m/z* calculated for C_52_H_39_N_3_O_2_S_2_H [M+H]^+^: 802.2562; found: 802.2563.

**vi: Synthesis of DTP-g**

In a dry 100 mL two-necked flask, DTP-Br (1.00 g, 1.59 mmol), 1*H*-benzo[*g*]indole (0.79 g, 4.77 mmol), Pd_2_(dba)_3_ (72.7 mg, 79.44 µmol), (*t*-Bu)_3_PHBF_4_ (91.2 mg, 317.75 µmol), and NaO*t*Bu (458.1 mg, 4.77 mmol) were dissolved in 20 mL of toluene under a nitrogen atmosphere. The reaction mixture was refluxed at 110 °C for 48 hours. After cooling to room temperature, the mixture was washed with brine. The organic layer was then extracted three times with ethyl acetate, and the combined organic extracts were dried over anhydrous Na_2_SO_4_. After solvent removal, the crude product was purified by column chromatography (CH_2_Cl_2_ / Petroleum ether, 1/1 v/v) to yield the desired product as a yellow-green solid (0.29 g, 22.8%).

^1^H NMR (500 MHz, CD_2_Cl_2_) δ 8.12 (d, *J* = 8.0 Hz, 4H), 7.95 (d, *J* = 8.1 Hz, 2H), 7.78 (d, *J* = 8.5 Hz, 2H), 7.74 (s, 2H), 7.72 (d, *J* = 8.0 Hz, 4H), 7.61 (d, *J* = 8.6 Hz, 2H), 7.58 (d, *J* = 8.5 Hz, 2H), 7.39 (t, *J* = 7.5 Hz, 2H), 7.31 (d, *J* = 3.1 Hz, 2H), 7.27 (t, *J* = 7.7 Hz, 2H), 6.86 (d, *J* = 3.1 Hz, 2H), 4.32 (t, *J* = 7.1 Hz, 2H), 1.92 (p, *J* = 7.1 Hz, 2H), 1.35 - 1.25 (m, 6H), 0.82 (t, *J* = 7.0 Hz, 3H).

^13^C NMR (126 MHz, CD_2_Cl_2_) δ 187.47, 147.39, 145.19, 143.40, 137.66, 131.55, 130.44, 130.02, 129.38, 129.09, 127.02, 126.59, 124.96, 123.76, 122.34, 122.15, 121.68, 121.14, 120.82, 118.20, 105.04, 47.58, 31.33, 30.28, 26.67, 22.51, 13.73.

MS (TOF MS AP+): *m/z* calculated for C_52_H_39_N_3_O_2_S_2_H [M+H]^+^: 802.2562; found: 802.2571.

**Figure S1.** ^1^H NMR spectrum of Lab Cz in DMSO.

**Figure S2.** ^13^C NMR spectrum of Lab Cz in DMSO.

**Figure S3.** ^1^H NMR spectrum of DTP in CD_2_Cl_2_.

**Figure S4.** ^13^C NMR spectrum of DTP in CD_2_Cl_2_.

**Figure S5.** MS spectrum of DTP.

**Figure S6.** ^1^H NMR spectrum of DTP-Br in CD_2_Cl_2_.

**Figure S7.** ^13^C NMR spectrum of DTP-Br in CD_2_Cl_2_.

**Figure S8.** MS spectrum of DTP-Br.

**Figure S9.** ^1^H NMR spectrum of DTP-Cz in CD_2_Cl_2_.

**Figure S10.** ^13^C NMR spectrum of DTP-Cz in CD_2_Cl_2_.

**Figure S11.** MS spectrum of DTP-Cz.

**Figure S12.** ^1^H NMR spectrum of DTP-g in CD_2_Cl_2_.

**Figure S13.** ^13^C NMR spectrum of DTP-g in CD_2_Cl_2_.

**Figure S14.** MS spectrum of DTP-g.

**Figure S15.** ^1^H NMR spectrum of DTP-f in CD_2_Cl_2_.

**Figure S16.** ^13^C NMR spectrum of DTP-f in CD_2_Cl_2_.

**Figure S17.** MS spectrum of DTP-f.

**Figure S18.** (a) Steady state PL spectra of DTP-Cz, DTP-g, and DTP-f in dilute toluene solution (monomer). (b) PL spectra in the powder state (aggregate), showing a huge red-shift compared to the dilute solution.

**Figure S19.** Normalized UV-vis absorption spectra of (a) DTP-Cz, (b) DTP-g, and (c) DTP-f in different solvents.

**Figure S20.** (a-c) Concentration-dependent PL spectra of DTP-Cz, DTP-g, and DTP-f doped in PMMA (0.01-10 wt.%), showing a gradual red-shift of emission toward that of the ground powder with increasing concentration. (d-f) Corresponding CIE 1931 chromaticity diagrams. Among the three, DTP-f exhibits the best miscibility in PMMA.

**Figure S21.** PL spectrum of pure PMMA film, showing negligible emission under the measurement conditions.

**Figure S22.** (a) Steady-state and (b) delayed PL spectra of DTP-Cz, DTP-g, and DTP-f doped in PMMA at 1 wt.%, showing prompt fluorescence (PF), delayed fluorescence (DF), and room temperature phosphorescence (RTP).

**Figure S23.** Overlap of prompt and delayed PL spectra of DTP-Cz doped in PMMA at (a) 0.01 wt.%, (b) 0.1 wt.%, and (c) 1 wt.%. The identical spectral shape and peak position of PF and DF confirm the delayed fluorescence origin. Both PF and DF exhibit red-shifts with increasing concentration, while the RTP wavelength remains essentially unchanged.

**Figure S24.** Overlap of prompt and delayed PL spectra of DTP-g doped in PMMA at (a) 0.01 wt.%, (b) 0.1 wt.%, and (c) 1 wt.%. The identical spectral shape and peak position of PF and DF confirm the delayed fluorescence origin. Both PF and DF exhibit red-shifts with increasing concentration, while the RTP wavelength remains essentially unchanged.

**Figure S25.** Photographs of DTP-Cz, DTP-g, and DTP-f crystals under daylight and UV irradiation (365 nm). Scale bar: 1 mm.

**Figure S26.** ORTEP diagrams of DTP-f, showing molecular conformation in the crystalline state. (CCDC 2549138)

**Figure S27.** Steady-state and delayed PL spectra of (a) DTP-Cz, (b) DTP-g, and (c) DTP-f doped in BPBr (0.1 wt.%), showing stronger RTP emission compared with PMMA doping system. The enhanced RTP indicates triplet-triplet energy transfer (TTET) from BPBr to the doped molecules.

**Figure S28.** Time-resolved decay curves of a) crystalline BPBr (melt-cast) monitored at 500 and 550 nm, b) DTP-f doped BPBr 1 wt.% monitored at 500, 550, 650, 700 nm and c) DTP-f doped BPBr 5 wt.% monitored at 650, 700 nm

**Figure S29.** Time-resolved decay curves of DTP-Cz doped in PMMA at different concentrations. (a) Decay monitored at the DF peak, showing shortened delayed lifetime with increasing concentration. (b) Decay monitored at the RTP peak, showing nearly unchanged phosphorescence lifetime.

**Figure S30.** Time-resolved decay curves of DTP-g doped in PMMA at different concentrations. (a) Decay monitored at the DF peak, showing shortened delayed lifetime with increasing concentration. (b) Decay monitored at the RTP peak, showing nearly unchanged phosphorescence lifetime.

**Figure S31.** Time-resolved decay curves of DTP-f doped in PMMA at different concentrations. (a) Decay monitored at the DF peak, showing shortened delayed lifetime with increasing concentration. (b) Decay monitored at the RTP peak, showing nearly unchanged phosphorescence lifetime.

**Figure S32.** (a-c) PL spectra of DTP-Cz, DTP-g, and DTP-f in THF/water mixtures with water volume fraction (*f_w_*) ranging from 0 to 99 vol%. (d-f) Corresponding *α*_AIE_ (*I/I_0_*, where *I_0_* is the emission intensity at *f_w_* = 0) and emission peak wavelength as a function of *f_w_*. All three compounds exhibit aggregation-caused quenching (ACQ) behavior.

**Figure S33.** (a) PL spectra of DTP-f in toluene/hexane mixtures with hexane volume fraction (*f_h_*) ranging from 0 to 99 vol%. (b) Corresponding *α*_AIE_ and emission peak wavelength as a function of *f_h_*.

**Figure S34.** (a) PL spectra of DTP-Cz, DTP-g, and DTP-f (10^-5^ M) in 2-MeTHF at room temperature; inset: photograph of the three solutions under UV irradiation. (b) Prompt emission spectra in 2-MeTHF at 77 K. (c) Delayed emission spectra of the 2-MeTHF solutions at 77 K with a delay time of 2 ms, showing phosphorescence-dominated emission.

**Figure S35.** Photographs of DTP-Cz (10^-5^ M) in 2-MeTHF at 77 K (liquid nitrogen) under UV (365 nm) irradiation and after turning off the UV light, showing time-dependent afterglow evolution.

Figure S36. Calculated T1→S0 transition characters of DTP-f in the monomeric and aggregated states, showing dominant localized 3LE character in both cases. This result supports the nearly unchanged RTP emission wavelength upon aggregation.

**Figure S37.** Temperature-dependent delayed emission properties of DTP-f doped in PMMA at 1 wt.%. (a) Enlarged delayed emission spectra in the 400–600 nm region recorded at different temperatures with a delay time of 1 ms, showing the thermally enhanced delayed fluorescence band. (b) Temperature-dependent delayed emission spectra in the 400–800 nm region recorded with a delay time of 1 ms. (c) Temperature-dependent time-resolved decay curves of DF. (d) Temperature-dependent time-resolved decay curves of RTP. The delayed fluorescence becomes more pronounced at elevated temperatures, whereas the RTP band is gradually quenched and shortened upon heating, indicating a thermally activated delayed fluorescence process competing with phosphorescence.

Table S1. Excitation-wavelength-dependent overall PLQY of DTP-f @ PMMA films with different doping concentrations.

Table S2. Overall PLQY of BPBr, DTP-f @ PMMA, and DTP-f @ BPBr systems under 300–450 nm excitation.

Table S3. Overall PLQY and qualitative DTP-f phosphorescence contribution under 400 nm excitation.

**Reference**

[1] Gaussian 16, Revision C.01, M. J. Frisch, G. W. Trucks, H. B. Schlegel, G. E. Scuseria, M. A. Robb, J. R. Cheeseman, G. Scalmani, V. Barone, G. A. Petersson, H. Nakatsuji, X. Li, M. Caricato, A. V. Marenich, J. Bloino, B. G. Janesko, R. Gomperts, B. Mennucci, H. P. Hratchian, J. V. Ortiz, A. F. Izmaylov, J. L. Sonnenberg, Williams, F. Ding, F. Lipparini, F. Egidi, J. Goings, B. Peng, A. Petrone, T. Henderson, D. Ranasinghe, V. G. Zakrzewski, J. Gao, N. Rega, G. Zheng, W. Liang, M. Hada, M. Ehara, K. Toyota, R. Fukuda, J. Hasegawa, M. Ishida, T. Nakajima, Y. Honda, O. Kitao, H. Nakai, T. Vreven, K. Throssell, J. A. Montgomery Jr., J. E. Peralta, F. Ogliaro, M. J. Bearpark, J. J. Heyd, E. N. Brothers, K. N. Kudin, V. N. Staroverov, T. A. Keith, R. Kobayashi, J. Normand, K. Raghavachari, A. P. Rendell, J. C. Burant, S. S. Iyengar, J. Tomasi, M. Cossi, J. M. Millam, M. Klene, C. Adamo, R. Cammi, J. W. Ochterski, R. L. Martin, K. Morokuma, O. Farkas, J. B. Foresman, D. J. Fox, Gaussian, Inc., Wallingford, CT, 2016.

[2] Adamo, C.; Barone, V. Toward Reliable Density Functional Methods Without Adjustable Parameters: The PBE0 Model. *J. Chem. Phys*. 1999, 110, 6158-6170.

[3] Grimme, S.; Antony, J.; Ehrlich, S.; Krieg, H. A Consistent and Accurate *ab initio* Parametrization of Density Functional Dispersion Correction (DFT-D) for the 94 Elements H-Pu. *J. Chem. Phys.* 2010, *132* (15), 154104.

[4] Humphrey, W.; Dalke, A.; Schulten, K. VMD: Visual molecular dynamics. *J. Mol. Graph. Model.* 1996, *14* (1), 33-38.

[5] Lu, T.; Chen, F. Multiwfn: A multifunctional wavefunction analyzer. *J. Comput. Chem.* 2012, *33* (5), 580-592.

[6] Neese, F. The ORCA program system. *WIREs Comput. Mol. Sci.* 2012, *2*, 73-78.
